# Model selection and averaging of nonlinear mixed-effect models for robust phase III dose selection

**DOI:** 10.1007/s10928-017-9550-0

**Published:** 2017-11-04

**Authors:** Yasunori Aoki, Daniel Röshammar, Bengt Hamrén, Andrew C. Hooker

**Affiliations:** 1 Department of Pharmaceutical Biosciences, Uppsala University, Uppsala, Sweden; 2 Present Address: National Institute of Informatics, Tokyo, Japan; 3 Quantitative Clinical Pharmacology, Innovative Medicines and Early Development, IMED Biotech Unit, AstraZeneca, Gothenburg, Sweden; 4 Present Address: SGS Exprimo, Mechelen, Belgium

**Keywords:** Model averaging, Model selection, Pharmacometrics, Phase IIb clinical trial, Dose finding study, Mathematical modelling, Dose–effect relationship

## Abstract

Population model-based (pharmacometric) approaches are widely used for the analyses of phase IIb clinical trial data to increase the accuracy of the dose selection for phase III clinical trials. On the other hand, if the analysis is based on one selected model, model selection bias can potentially spoil the accuracy of the dose selection process. In this paper, four methods that assume a number of pre-defined model structure candidates, for example a set of dose–response shape functions, and then combine or select those candidate models are introduced. The key hypothesis is that by combining both model structure uncertainty and model parameter uncertainty using these methodologies, we can make a more robust model based dose selection decision at the end of a phase IIb clinical trial. These methods are investigated using realistic simulation studies based on the study protocol of an actual phase IIb trial for an oral asthma drug candidate (AZD1981). Based on the simulation study, it is demonstrated that a bootstrap model selection method properly avoids model selection bias and in most cases increases the accuracy of the end of phase IIb decision. Thus, we recommend using this bootstrap model selection method when conducting population model-based decision-making at the end of phase IIb clinical trials.

**Electronic supplementary material:**

The online version of this article (doi:10.1007/s10928-017-9550-0) contains supplementary material, which is available to authorized users.

## Introduction and background

Quantifying the probability of achieving the targeted efficacy and safety response is crucial for go/no-go investment decision-making in a drug development program. This is particularly crucial when analyzing phase IIb (PhIIb) dose-finding studies to select the phase III dose(s) given the costs of phase III studies.

It has previously been shown that population model-based (pharmacometric) approaches can drastically increase the power to identify drug effects in clinical trial data analysis compared to conventional statistical analysis (e.g., [1]). On the other hand, the model-based approach can be hindered by model selection bias if a single model structure is assumed and used for the analysis (e.g., [2, 3]). There have been several attempts through model averaging and model selection to weaken the model structure assumptions by considering multiple possible model candidates in the analysis [4–9].

In this paper, we introduce four methods that assume a number of pre-defined model candidates and then combine or select those candidate models in different ways to make predictions and to account for uncertainty in those predictions. The first method is “simple” model selection where a set of model structures are pre-specified and a model is chosen according to a statistical criterion. Uncertainty in model prediction is then derived from parameter uncertainty, based on a bootstrap procedure using the selected model. The second method is a bootstrapped model selection procedure, where, for each bootstrap dataset, the best-fit of the candidate models is chosen according to a statistical criterion. Simulation from each bootstrap selected model with its optimal parameter will then generate a distribution of the quantities of interest, accounting for both model and parameter uncertainty (similar methods can be found in the literature, e.g., [11, 12]). The third method is a conventional model averaging procedure where each candidate model is fit to the data and uncertainty is quantified via bootstrap. Simulations (including parameter uncertainty) from each candidate model of the distributions of the quantities of interest are then combined as a weighted average depending on model fit to the original data. The fourth method is a bootstrapped model averaging procedure, where the weighting for the weighted average calculations are based on model fit to each bootstrapped dataset (as opposed to the model-fit to the original data).

Comparison of these methods and a standard statistical method (pair-wise ANOVA and the groupwise estimate of an average change from baseline) are done using clinical trial simulations of dose-finding studies. To make the simulations as realistic as possible, we have based them on the protocol of an actual PhIIb trial for an oral asthma drug candidate (AZD1981) as well as the data from the placebo arm of that trial. Drug effects using various model structures were simulated for five different dose arms (placebo plus four active arms). The different analysis methods were then used to calculate the probability of achieving target endpoint and then choose the minimum effective dose (MED).

## Methods

### Phase IIb dose-finding case study

Part of the PhIIb clinical trial data and the study protocol of the asthma drug candidate AZD1981 (ClinicalTrials.gov/NCT01197794) was utilized in this work. One endpoint goal of the study was to demonstrate that the drug improved the forced expiratory volume in 1 s (FEV1) of asthma patients by, on average, at least 0.1 L (placebo and baseline adjusted). This clinical trial was chosen as a case study since FEV1 is a highly variable endpoint (standard deviation of 0.3 L in the placebo effect) relative to the expected effect magnitude; hence it is hard to characterize the dose–effect relationship from PhIIb clinical trials.

This study was conducted for 12 weeks and FEV1 was measured every 2 weeks (for a total of 7 measurements, or visits). The first measurement was a screening visit and the second measurement was used as a baseline measurement after which either placebo, AZD1981 10, 20, 100 or 400 mg was administered twice daily (bid).

The data from the placebo group and the lowest dose group of the PhIIb clinical trial for AZD1981 was provided for this analysis. Dosing information was not provided; however, as there were no statistically significant differences between the placebo group and the lowest dose group as described in [13], in this paper we refer this dataset as a “placebo” dataset. This dataset comprises 324 patients with a total of 1803 FEV1 measurements.

### Models

#### Placebo model

The following placebo model was developed using the placebo dataset from the PhIIb clinical trial for AZD1981:

$$\begin{aligned} {\text{FEV}}1 & = \left( {{\text{FEV}}1_{\text{Baseline}} + \left\{ {\begin{array}{*{20}l} 0 \hfill & {{\text{if visit}} = 1,2} \hfill \\ {{\text{FEV}}1_{\text{Placebo}} } \hfill & {{\text{if visit}} = 3,4,5,6,7} \hfill \\ \end{array} } \right\}} \right) \cdot (1 + \epsilon_{1} ) + \epsilon_{2} \\ {\text{FEV}}1_{\text{Placebo}} & = \theta_{1} + \eta_{1} \\ {\text{FEV}}1_{\text{Baseline}} & = \theta_{2} e^{{\eta_{2} }} \\& \quad \times (1 + \theta_{3} ({\text{FEV}}1_{{{\text{\% of normal}}}} - \overline{{{\text{FEV}}1}}_{\text{\% of normal}})) \\& \quad \times (1 + \theta_{4} ({\text{Age}} - \overline{\text{Age}} )) \\& \quad \times \left\{ {\begin{array}{*{20}l} 1 \hfill & {\text{if Male}} \hfill \\ {\theta_{5} } \hfill & {\text{if Female}} \hfill \\ \end{array} } \right. \\ \eta_1 & \sim {\mathcal{N}}(0,\omega_{1}^{2} ) \\ \eta_2 & \sim {\mathcal{N}}(0,\omega_{2}^{2} ) \\ \epsilon_1 & \sim {\mathcal{N}}(0,\sigma_{1}^{2} ) \\ \epsilon_2 & \sim {\mathcal{N}}(0,\sigma_{2}^{2} ) \\ \end{aligned}$$

where ${\text{FEV}}1_{{{\text{\% of normal}}}}$ is the percentage of FEV1 at visit 2 compared to the predicted normal and $\overline{{{\text{FEV}}1}}_{\text{\% of normal}}$ is its population mean, $\overline{\text{Age}}$ is the mean of the age of the patients. All the estimated model parameters can be found in Table 1.

**Table 1:** Estimated Parameters of the placebo model of FEV1 of Asthma patients based on the placebo and lowest dose group of the PhIIb clinical trial for AZD 1981

Model parameter	Description	Estimated value (RSE%)
$\theta_{1}$	Placebo effect	0.169 L (11.9)
$\theta_{2}$	Baseline	2.51 L (0.828)
$\theta_{3}$	Covariate effect of ${\text{FEV}}1_{{{\text{\% of normal}}}}$	0.0129 L^−1^(3.87)
$\theta_{4}$	Covariate effect of age	−0.0105 year^−1^ (5.01)
$\theta_{5}$	Covariate effect of sex	0.719 (1.43)
$\omega_{1}$	SD of IIV of the placebo effect	0.303 L (8.88)
$\omega_{2}$	SD of IIV of the baseline	0.105 (5.97)
$\sigma_{1}$	SD of proportional RUV	0.0832 (7.60)
$\sigma_{2}$	SD of additive RUV	0.102 L (18.8)

*IIV* inter-individual variability, *RUV* residual unexplained variability, *SD* standard deviation, *RSE* the relative standard error was approximated using a variance–covariance matrix, and the computational result was verified using preconditioning [19]

Previously Wang et al. [14] have modelled a placebo effect on the FEV1 measurement. The model presented here differs slightly from Wang et al. in that this model employs a step function for the placebo effect model with respect to visit, while Wang et al. have used exponential models with time as the independent variable. Wang et al. state that the placebo effect plateaus at 0.806 ${\text{week}}^{ - 1}$ while the current dataset contains FEV1 measurements only every 2 weeks; hence the rate constant of the exponential model was not estimable from this dataset.

#### Drug effect models

In this work, we simulate and estimate using a number of different dose–effect relationships DE_j_:

$$\begin{aligned} {\text{DE}}_{0} &= 0\; ( {\text{no treatment effect)}} \hfill \\ {\text{DE}}_{1} ({\text{dose}};p_{1} ) &= p_{1} \cdot {\text{dose}}\; ( {\text{linear model)}} \hfill \\ {\text{DE}}_{2} ({\text{dose}};p_{1} ,p_{2} ) &= p_{1} \cdot { \log }(1 + p_{2} {\text{dose}})\; ( {\text{log-linear model)}} \hfill \\ {\text{DE}}_{3} ({\text{dose}};{\text{EMAX}},{\text{EC}}50) &= {\text{EMAX}} \cdot \frac{\text{dose}}{{{\text{EC}}50 + {\text{dose}}}}\; ( {\text{Emax model)}} \hfill \\ {\text{DE}}_{4} ({\text{dose}};{\text{EMAX}},{\text{EC}}50,\gamma ) &= {\text{EMAX}} \cdot \frac{{{\text{dose}}^{\gamma } }}{{{\text{EC}}50^{\gamma } + {\text{dose}}^{\gamma } }}\; ( {\text{sigmoid Emax model)}} \hfill \\ \end{aligned}$$

To create simulated datasets, we add different simulated drug effects, with different parameters, using the above models, to the FEV1 measurements of the placebo data (more detail below). For estimation using the model-based analysis methods described below, we embed these dose–effect relationships into the placebo model as follows:

$${\text{FEV}}1_{j} = \left( {{\text{Baseline}} + {\text{PlaceboEffect}} + {\text{DE}}_{j} } \right) \cdot (1 + \epsilon_{1} ) + \epsilon_{2}$$

### Analysis methods

#### Statistical analysis used for the PhIIb clinical trial for AZD1981

The primary statistical analysis of the data from the PhIIb clinical trial for AZD1981 to determine the MED was performed using a pair-wise ANOVA and a group wise estimate of treatment effect. Briefly, the treatment effect was measured as the change from baseline (average of all available data from visits 3–7 minus baseline) per dose group. The MED was identified via a two-stage step-down procedure to select either 400, 100, 40, 10 mg or “stop” (do not proceed to phase III). The procedure was as follows: (1) starting with the highest (400 mg) dose-arm conduct a one-sided ANOVA comparison with the placebo-arm. (2) If the difference is significant (significance level of 0.05) check that the average treatment effect in the arm is greater than the primary efficacy variable (0.1 L). (3) If both steps 1 and 2 are satisfied then proceed to the next dose dose-arm (100 mg) and repeat, otherwise move to step 4. (4) Choose the lowest dose arm where both steps 1 and 2 are satisfied (Note that if 100 mg satisfies steps 1 and 2 but 40 mg does not then the MED will be 100 mg in this process, even if 10 mg might also satisfy steps 1 and 2).

#### Model selection and averaging analysis methods

Below is an overview of four methods that assume a number of pre-defined model candidates and then combine or select those candidate models in different ways to make predictions and to account for uncertainty in those predictions. For the given example, the methods were meant to compare with the standard determination of the MED, identified in the original study using the two-stage step-down procedure described earlier in this section. Thus, in the following methods, there should be a test for drug effect as well as a determination if that effect is greater than a given minimum effect size. In all methods, the test for drug effect is done using a likelihood ratio test (LRT) against the placebo model (5% significance level) [8]. Determination of effect sizes at specific doses is done by first computing the change from baseline average (population mean) effect size, and uncertainty around that effect size, predicted by the different methods described below, for a given dose. MED is then chosen as the lowest studied dose with a predefined probability to be above a target effect. For a full technical description of the methods, we refer the readers to the Appendix. The methods below are previously presented by the authors as a conference contribution [9], and Method 3 was presented at an earlier conference [10].

#### Method 1: model selection

1. Fit each candidate model structure to the original data and estimate the model parameters and maximum likelihood (see Fig. 1).

**Fig. 1:**
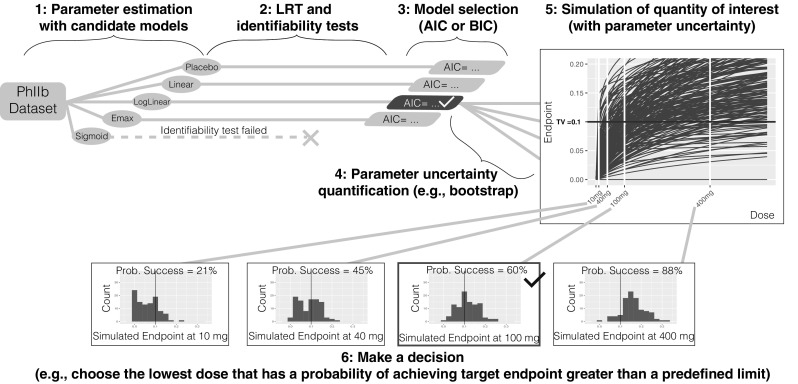
Method 1 model selection
2. For each candidate model, perform a numerical identifiability test (see appendix for detail) and LRT against the placebo model and reject any model structure that fails either of these tests.
3. Select one model structure among the remaining model candidates using a statistical criterion based on the maximum likelihood (e.g., the Akaike information criterion, AIC, the Bayesian information criterion, BIC, etc.).
4. Quantify parameter uncertainty using case sampling bootstrap and the selected model structure.
5. Simulate the quantities of interest (with uncertainty); in this case, the dose-endpoint (change from baseline population mean effect size) relationships using the selected model structure and model parameters obtained from the bootstrap procedure.
6. Make a decision; in this case, choose the lowest dose (given allowed dose levels) that has a probability of achieving target endpoint greater than a predefined limit.

#### Method 2: bootstrap model selection

1. Create bootstrap datasets based on the original data using a case sampling bootstrap procedure (see Fig. 2).

**Fig. 2:**
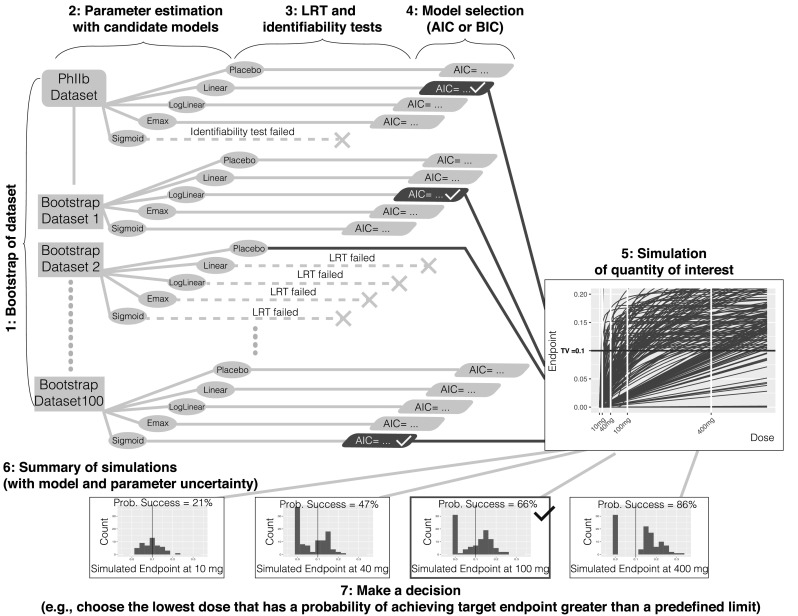
Method 2 bootstrap model selection
2. For each bootstrap dataset estimate parameters and the maximum likelihood for each candidate model structure.
3. For each bootstrap dataset, and for each candidate model, perform a numerical identifiability test and LRT against the placebo model and reject any model structure that fails either of these tests.
4. For each bootstrap dataset, select one model structure among the remaining model candidates using a statistical criterion based on the maximum likelihood (e.g., AIC, BIC, etc.).
5. For each bootstrap dataset, simulate the quantities of interest; in this case, the dose-endpoint (change from baseline population mean effect size) relationships using the selected model structure and model parameters obtained from that bootstrap dataset.
6. Summarize the simulations; in this case, compute the probability of achieving the target endpoint at each dose of interest using the simulated dose-endpoint relationships.
7. Make a decision; in this case, choose the lowest dose (given allowed dose levels) that has a probability of achieving the target endpoint greater than a predefined limit.

#### Method 3: model averaging

1. Fit each candidate model structure to the original data and estimate model parameters and maximum likelihood (see Fig. 3).

**Fig. 3:**
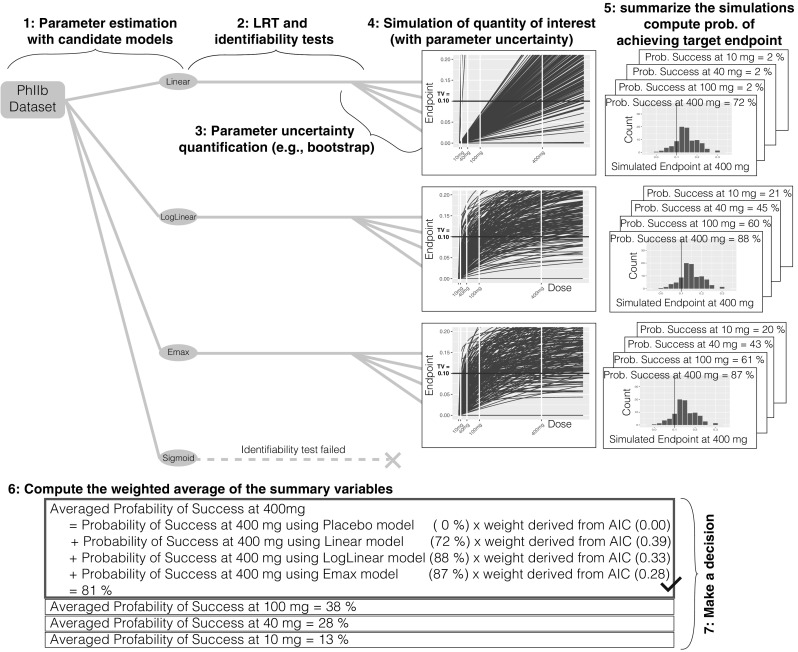
Method 3 model averaging
2. For each candidate model, perform a numerical identifiability test and LRT against the placebo model and reject any model structure that fails either of these tests.
3. For each model structure, quantify parameter uncertainty using case sampling bootstrap methodology to obtain the distribution of the model parameters.
4. For each model structure, simulate the quantities of interest; in this case, the dose-endpoint relationships using the model parameters obtained from the bootstrap method.
5. For each model structure, summarize the simulations; in this case, compute the probability of achieving the target endpoint at each dose of interest using the simulated dose-endpoint relationships.
6. Compute the weighted average of the summary variables obtained in step 5; in this case, the probability of achieving the target endpoint at each dose over the model structures, where the weights are derived from the maximum likelihood obtained in step 1 (e.g., AIC, BIC, etc.).
7. Make a decision; in this case, choose the lowest dose (given allowed dose levels) that has a probability of achieving target endpoint greater than a predefined limit.

#### Method 4: bootstrap model averaging

1. Create bootstrap datasets based on the original data using a case sampling bootstrap procedure (see Fig. 4).

**Fig. 4:**
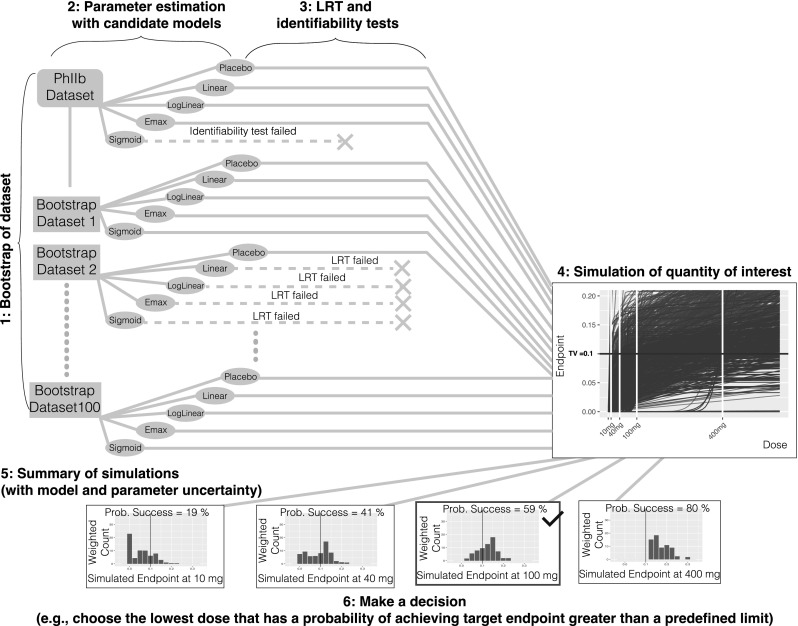
Method 4 bootstrap model averaging
2. For each bootstrap dataset estimate parameters and the maximum likelihood for each candidate model structure.
3. For each bootstrap dataset, and for each candidate model, perform a numerical identifiability test and LRT against the placebo model and reject any model structure that fails either of these tests.
4. For each bootstrap dataset and each model structure, simulate the quantities of interest; in this case, the dose-endpoint (change from baseline population mean effect size) relationships using the selected model structure and model parameters obtained from that bootstrap dataset.
5. Summarize the simulations; in this case, compute the weighted average of the probability of achieving the target endpoint at each dose using the dose-endpoint relationships for all the model structures and all the bootstrap datasets (except the ones that failed the LRT or identifiability test). The weights are derived from the maximum likelihood obtained in step 2 (using AIC, BIC, etc.).
6. Make a decision; in this case, choose the lowest dose (given allowed dose levels) that has a probability of achieving the target endpoint greater than a predefined limit.

### Single model based approach

To compare the proposed methods against the idealized situation where the underlining true model structure is known before the analysis, we compare with a single model based approach where the model used to analyze the dataset is the same as the model used to simulate that dataset. Note that this single model based analysis using the simulation model is an idealistic scenario. In a real PhIIb dataset analysis (i.e., when analyzing data that was not simulated) it is not realistic to assume the exact underlying model structure is known a priori. The method has the following steps:

1. Perform LRT between the model with and without drug effect. If the model does not pass the LRT, make a “stop” decision.
2. If the model with drug effect passes the LRT, estimate the parameter uncertainty using a case sampling bootstrap.
3. Simulate the quantities of interest (with uncertainty); in this case, the dose-endpoint (change from baseline population mean effect size) relationships using the model parameter distribution obtained from the bootstrap procedure.
4. Make a decision; in this case, select the dose based on the required probability of achieving the target endpoint.

### Numerical experiments

To test the proposed model averaging and selection methodologies, we have simulated dose-finding studies under various designs and experimental scenarios. All numerical computations were done using NONMEM [15] version 7.3, PsN [16] version 4.6 on a Linux Cluster, with Intel Xeon E5645 2.4 GHz processors, 90 GB of memory, Scientific Linux release 6.5, GCC 4.4.7 and Perl version 5.10.1. To assure reproducibility of the numerical experiments we had a fixed random seed when the bootstrap method was performed using PsN. All computation outside of NONMEM and PsN was done using R version 3.2 [17] and all plots are made using ggplot2 [18].

#### Simulation studies based on placebo data

To create simulated datasets, we have simply added different simulated drug effects to the FEV1 measurements of the placebo data. We have randomly generated the artificial drug effect so that the theoretical minimum effective dose (tMED, i.e., the exact dose that achieves a drug effect of 0.1 L) is uniformly distributed in the ranges shown in Table 2.

**Table 2:** Various scenarios of the simulation studies

	Theoretical minimum effective dose (tMED)	Correct dose finding/decision
Simulation Study 1	0–10 mg	10 mg
Simulation Study 2	10–40 mg	40 mg
Simulation Study 3	40–100 mg	100 mg
Simulation Study 4	100–400 mg	400 mg
Simulation Study 5	No drug effect	Stop

For each Simulation Study 1–5, we have constructed 300 PhIIb clinical trial simulation datasets (1500 datasets in total). Simulation Studies 1–4 are constructed to test each analysis method for the accuracy of finding tMED, while Simulation Study 5 is constructed to test each method for the accuracy of Type-1 error control.

In each Simulation study, log-linear, emax and sigmoidal models (described above) were used to simulate the drug effects (DE_j_) (100 datasets each for each of three model structures, hence 300 total simulated datasets in one simulation study). For each data set, we first randomly choose tMED in the range shown in Table 2. Then the model parameters are chosen randomly as follows:

For the log-linear model, $p_{1}$ and $p_{2}$ are chosen so that

$$\begin{aligned} {\text{DE}}_{2} (1000,p_{1} ,p_{2} )&\sim {\text{unif}}(0.2,0.3) \hfill \\ {\text{DE}}_{2} ({\text{tMED}};p_{1} ,p_{2} ) &= 0.1 \hfill \\ \end{aligned}$$

For the emax model, ${\text{EMAX}}$ and ${\text{EC}}50$ are chosen so that

$$\begin{aligned} {\text{EMAX}}&\sim {\text{unif}}(0.2,0.3) \hfill \\ {\text{DE}}_{3} ({\text{tMED}};{\text{EMAX}},{\text{EC}}50) &= 0.1 \hfill \\ \end{aligned}$$

For the sigmoidal model, ${\text{EMAX}}$, ${\text{EC}}50$ and $\gamma$ are chosen so that

$$\begin{aligned} {\text{EMAX}}&\sim {\text{unif}}(0.2,0.3) \hfill \\ \gamma &\sim {\text{unif}}(0.5,4) \hfill \\ {\text{DE}}_{4} ({\text{tMED}};{\text{EMAX}},{\text{EC}}50,N) &= 0.1 \hfill \\ \end{aligned}$$

**Fig. 5:**
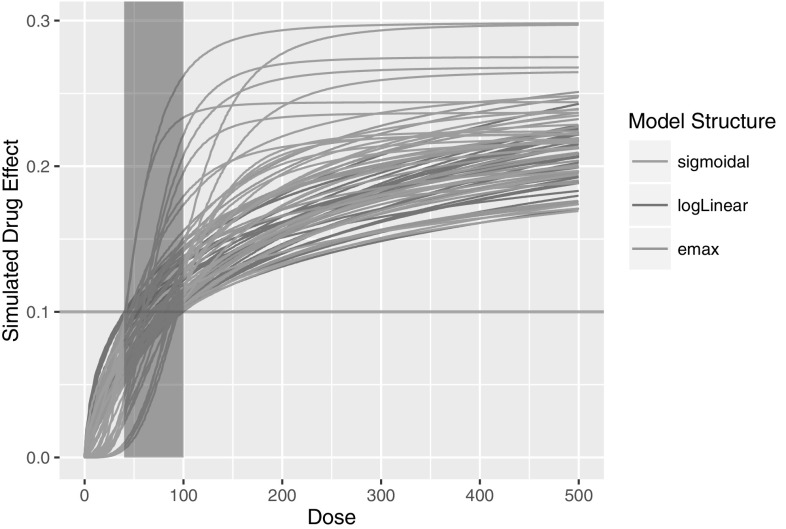
Plot of (some of) the simulated drug effect for Simulation Study 3. The theoretical minimum effective dose (the exact dose that achieves the target endpoint of 0.1 L) ranges between 40 and 100 mg hence the 100 mg dose is the correct dose selection for this simulation study

Note that to determine the parameters $p_{1}$ and $p_{2}$ for the log-linear model, we need to solve a nonlinear equation numerically and we do so by using the *uniroot* function in R. As can be seen in Fig. 5, we can create diverse realistic simulated drug effects by the above choice of model parameters while the range of tMED is constrained.

### Numerical experiment 1: dose finding accuracy

The simulated data from Simulation Studies 1–4 (when a drug effect is present) was analyzed using the methods presented above to determine the dose finding accuracy of the methods. Each method was used to find the MED for each trial simulation dataset and the probability of finding the correct dose was calculated (see Table 2).

For the model-based approaches, the MED dose was chosen as the minimum dose arm (of the investigated doses) with more than a 50% probability of achieving the target endpoint. 50% was chosen to match the statistical analysis used for the PhIIb clinical trial for AZD198, which evaluates if the average treatment effect in a dose arm is greater than the primary efficacy variable.

### Numerical experiment 2: type-1 error control accuracy

All methods presented above were used to determine the MED based on the data from Simulation Study 5 (the simulation study without simulated drug effect) to test the type-I error rate of the proposed methods. That is, the probability of choosing the MED to be either 10, 40, 100, or 400 mg while there is no simulated drug effect. The MED selection using the model-based approaches were determined at a 50% confidence level to fairly compare the method with the pairwise ANOVA method.

### Numerical experiment 3: decision-making accuracy

In the previous two numerical experiments the MED using the model-based approaches are determined at a 50% confidence level to fairly compare the method with the pairwise ANOVA method. However, in reality, more than 50% certainty may be desired when making a decision about which dose to use in a phase III trial [20]. For example, from an investment perspective, it may be more crucial to reduce the risk of proceeding to a phase III trial with insufficient effect than to determine the exact MED of a drug.

For this experiment, we define the “correct” decision to be when any dose higher than the theoretical MED is selected. For example, for Simulation Study 3 ($40\;{\text{mg}} < {\text{tMED}} \le 100\;{\text{mg}}$), if either 100 or 400 mg is chosen then the correct decision was made; while if dose 10 or 40 mg is chosen, or a “stop” decision is made, then the incorrect decision was made. Each method was then used to find the MED (70% confidence level for the model-based approaches) for each simulated dataset from Simulation Studies 1–4 (when a drug effect is present). The probability of each method making the correct decision was then calculated.

### Numerical experiment 4: probability of achieving target endpoint estimation accuracy

In the model averaging and selection methods investigated here, the dose selection is based on the probability of achieving the target endpoint, hence, accurate estimation of this probability is crucial. In this experiment, we investigate this probability estimation for each simulated dataset from Simulation Studies 1–4 (when a drug effect is present) in the following manner:

1. Select a predefined limit, *p*, for the probability of achieving the target effect.
2. Allow any dose (any positive real number) to be selected (not just the investigated dose levels) and choose the dose that is estimated to achieve the target endpoint with probability $p$ using the proposed model-based methods.
3. Repeat steps 1 and 2 for all 1200 simulated phase IIb datasets and count the number of times a dose above the theoretical minimum effective dose (tMED) is selected, from which the empirical probability of achieving the target effect is calculated.
4. Repeat steps 1–3 for $p = 0.01, 0.02, \ldots , 0.99$.

Note that if a method estimates the probability of achieving the target endpoint without bias, then the selected doses should be above tMED with probability $p$.

## Results

To concisely present the results for each of Numerical Experiments 1, 3, and 4, we has combined the results of Simulation Studies 1–4. Hence, for those experiments, the results are based on 1200 PhIIb clinical trial simulations. We refer the readers to the Appendix for a detailed discussion of the result for each simulation study. Further, the uncertainty of the numerical experiments has been quantified by randomly sampling trial simulations with replacement (1200 trial simulations for Numerical Experiments 1, 3, and 4, and 300 trial simulations for Numerical Experiment 2) and repeated the numerical experiments. For example, for Numerical Experiments 1, 3, and 4, 1200 trial simulations were sampled with replacement 100 times to produce 100 sets of the 1200 trial simulations. For each set of trial simulations, the numerical experiments were performed.

### Numerical experiment 1: dose finding accuracy

**Fig. 6:**
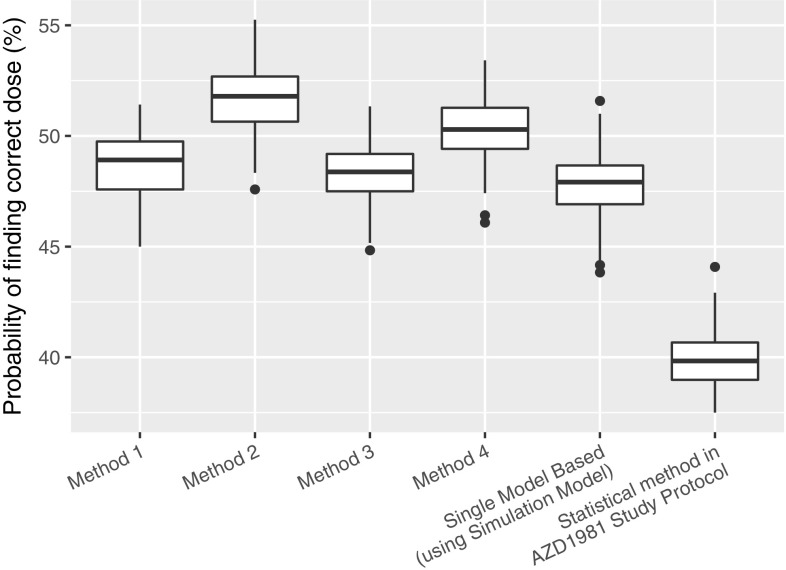
Probability of finding the correct dose. The edges of the boxes are 75th and 25th percentiles. The line in the box is the median and the whiskers extend to the largest and the smallest value within 1.5*inter-quartile range. Dots are the outliers outside of the whiskers

The dose finding accuracy of the various investigated methods is presented in Fig. 6. As can be seen, all the model based methods could find the correct dose more often than the statistical method used in the PhIIb AZD1981 study protocol. In addition, we can see that Methods 2 and 4 outperform Methods 1 and 3 and the Single Model Based approach (using the simulation model).

### Numerical experiment 2: type-1 error control accuracy

**Fig. 7:**
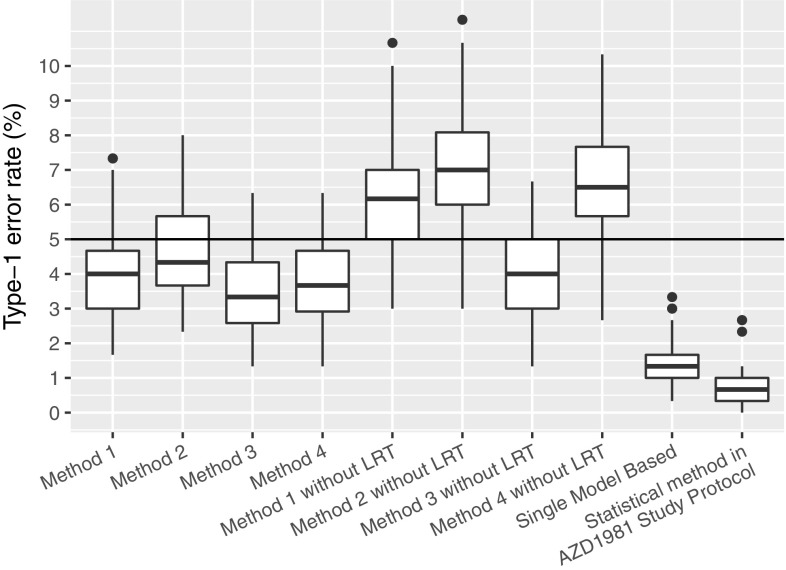
Type-1 error rate, the probability of choosing either 10 mg, 40 mg, 100 mg, or 400 mg while there is no simulated drug effect. The significance level of all the methods was set to 0.05 hence if the Type-1 error is correctly controlled the Type-1 error rate should be at 5% (indicated by the horizontal line)

The Type-I error control of the various investigated methods is presented in Fig. 7. As can be seen, Methods 1–4 control the type-I error accurately. Furthermore, we can see that the LRT is necessary for Methods 1, 2, and 4 to properly control the Type-1 error. Lastly, we see that the type-I error is lower than expected for the standard statistical test and Single Model Based method (using the Simulation Model).

### Numerical experiment 3: decision-making accuracy

**Fig. 8:**
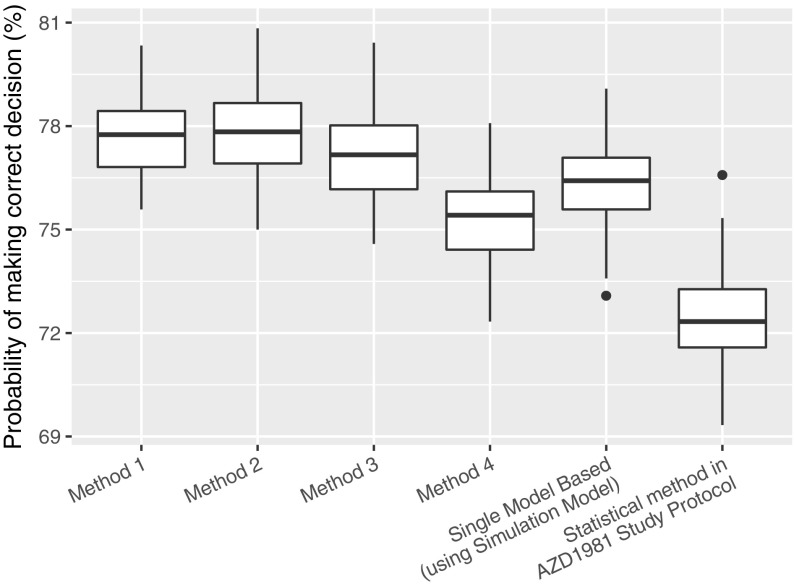
Probability of making the correct decision (the correct decision is defined as choosing the dose that is above tMED)

The decision-making accuracy of the various investigated methods is presented in Fig. 8. As can be seen, all model based method (Methods 1–4 and the Single Model Based method) makes the correct decision more often than the Statistical method employed in the AZD 1981 study protocol. Also, we can see that Method 4 performs relatively poorly compared to Methods 1–3.

### Numerical experiment 4: probability of achieving target endpoint estimation accuracy

**Fig. 9:**
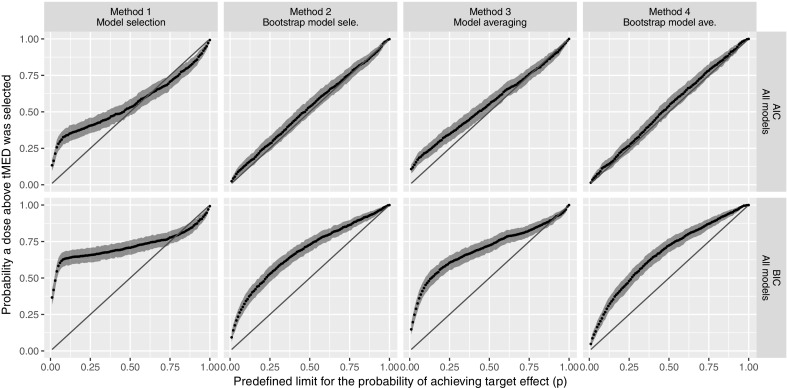
The accuracy of the calculated probability of achieving a target endpoint. The x-axis is the predefined limit for the probability of achieving target endpoint where the dose was chosen. The y-axis is the probability that the chosen dose by the various methods is above tMED. If the probability of achieving the target endpoint is estimated without bias, the plot should lie on the line of identity (red straight line). Grey shaded areas are 95% confidence intervals calculated by the random sampling with replacement of the 1200 trial simulation datasets

The Probability of achieving target endpoint estimation accuracy of the various investigated model-based methods is presented in Fig. 9. Note that if the investigated method estimates the probability of achieving the target endpoint without bias then the QQplot in Fig. 9 should follow the line of unity.

As can be seen in Fig. 9, Methods 2 and 4, using AIC as the statistical criteria in the methods, can calculate the probability of achieving target endpoint accurately. The bias on the calculated probability of achieving the target endpoint of the conventional model selection method (Method 1) is clearly observed. As discussed in literature (e.g., [2, 3]), if model selection is made based on one dataset the bias in the model selection procedure will be carried forward to subsequent analyses and any resulting quantity may be biased. Although the regular model averaging method (Method 3) should significantly decrease the effect of model selection bias, we still observe the presence of bias. Lastly, we observed that AIC is a more suitable statistical criterion than BIC for the proposed model averaging and selection methods.

## Discussion

This work presents model averaging and selection methods that incorporate both model structure and parameter estimation. We have tested the proposed methods through realistic PhIIb dose finding and decision-making scenarios and demonstrated that the proposed methods could help increase the overall probability of making the correct decision at the end of PhIIb studies.

Through all the numerical experiments, the model based approaches (Methods 1–4 and Single Model Based method) outperformed the pairwise ANOVA based method used in the AZD1981 study protocol. Numerical Experiments 1 and 4 have shown that Methods 2 and 4 perform better than other methods for finding MED and estimating probability of achieving endpoint. Numerical Experiment 3 has shown that Method 2 can be used to make the investment decision more accurately than Method 4. Experiment 2 has shown that Type-1 error can be appropriately controlled using the LRT and the Type-1 error control of Method 2 is marginally better than the other methods (Method 1, 3 and 4). Thus, within the scope of our numerical experiment, Method 2 was the most accurate and precise compared to the other tested methods.

The numerical experiments indicated that AIC is a more suitable statistical criterion than BIC for the model averaging and selection methods we have tested. BIC takes the number of observations into account when weighing the penalty for the extra degrees of freedom. For nonlinear mixed effect models, the informativeness of the dataset not only depends on the number of observations but also a number of individuals. Hence, we conjecture that, by naively using the number of observations, BIC does not properly weigh the penalty term and some other way of quantifying the ‘informativeness of observations’ is necessary.

Although we have conducted a wide-range of numerical experiments within the scope of this project, we believe the accumulation of more experiences of these and other methods through applying them to more scenarios would be desirable. For example, it would be interesting to compare and/or integrate the methods presented here with the MCP-Mod approach [21, 22]. The MCP-Mod methodology allows model averaging and selection methods for the “Mod” portion of that framework, but entails an initial multiple comparison procedure (the “MCP” portion) that may be redundant with the LRT used here. To promote the application and further development of the proposed methodologies, we have made the methodologies investigated here available as a GUI based open source software (available at www.bluetree.me and the Mac App Store, app name: modelAverage) as well as an R script supplied as the supplementary material of this paper.

## Conclusion

We recommend the use of the bootstrap model selection method (Method 2) presented in this paper when conducting model-based decision-making at the end of phase IIb study. The studies here indicate the proposed method reduces the analysis bias originating from model selection bias of single model structure based analyses. As a consequence of including model structure uncertainty, the quantified uncertainty may appear to be larger than single model based uncertainty; however, the method appears to more accurately reflect the true uncertainty of the investigated models and estimated parameters. The proposed method increases the probability of making the correct decisions at the end of phase IIb trial compared to conventional ANOVA-based Study Protocols.

## Appendix: Detailed description of the method

In this section, we present step by step explanations of our model selection and averaging methodologies. We have implemented this methodology in the C++ language, and an open source software with a graphical user-interface is available at www.bluetree.me and the Mac App Store (app name: modelAverage). Also, easy to read (computationally not optimized) R script used for the numerical experiments presented in this is available as the supplementary material.

### Electronic supplementary material

Below is the link to the electronic supplementary material. the following two supplementary materials are missing from the list below: AOKI_etal_ModelAveraging_RScript.docx , AOKI_etal_ModelAveraging_RScript.rmd

Supplementary material 1 (CSV 2 kb)

Supplementary material 2 (CSV 4 kb)

Supplementary material 3 (CSV 5 kb)

Supplementary material 4 (CSV 5 kb)

Supplementary material 5 (CSV 7 kb)

Supplementary material 6 (CSV 2 kb)

Supplementary material 7 (CSV 3 kb)

Supplementary material 8 (CSV 4 kb)

Supplementary material 9 (CSV 4 kb)

Supplementary material 10 (CSV 4 kb)

Supplementary material 11 (DOCX 62 kb)

Supplementary material 12 (RMD 23 kb)
