# Supplementary material for: Model selection and averaging of nonlinear mixed-effect models for robust phase III dose selection
Source: J Pharmacokinet Pharmacodyn. 2017 Nov 4;44(6):581–97. doi: 10.1007/s10928-017-9550-0 (PMC5686275; doi:10.1007/s10928-017-9550-0)

# R script

An example of implementing the proposed method in R script language is presented here. Prior to running this script, we have conducted case sampling bootstrap analysis using PsN and the result files are saved as "raw_results_estimation_model<modelNumber>.csv". Note that it is essential to use the same random seed for the bootstrap sampling for all models so that the bootstrap datasets created by PsN is the same for all models, e.g.,

bootstrap -seed=0 model0.mod
bootstrap -seed=0 model1.mod
bootstrap -seed=0 model2.mod
bootstrap -seed=0 model3.mod
bootstrap -seed=0 model4.mod

Also the bootstrap with preconditioned analysis results are saved in "precond_raw_results_estimation_model<modelNumber>.csv".

In each row_results file, the estimates of the model parameters from bootstrap datasets are stored. These estimated parameters can be found in the column with the name same as the name of the parameters. The -2 log likelihood is stored in the column named 'ofv'. For example, the following R script will store the bootstrap parameter estimates of $p_{1}$ of Model 2 in the array named 'model2_p1_parameters' and - 2 log likelihood in 'model2_ofv':

rawres_model1=read.csv("raw_results_estimation_model1.csv", header = TRUE, sep = ",")
 model2_p1_parameters=rawres_model1$p_1
 model2_ofv=rawres_model1$ofv

The csv files supplied with this supplementary document are the trimmed down version of the original raw_result files from the PsN bootstrap that only contains the ofv and parameter values of interest with first 100 bootstrap samples.

The following R script combines the bootstrap analyses results of various model structures and calculates the probability of achieving target endpoint for each study dose. Then chooses the minimum dose that achieves the target endpoint with predefined limit for the probability of achieving target endpoint. At the end two plots are made to visually represent the results.

## Set the parameters for the method

dose <- c(0, 10, 40, 100, 400) # \vec{x}_1 in the manuscript
 TV=0.1 # Target Value
 # if 1 identifiability of the parameter is checked usijng precond bootstrap raw result files.
 method=2 # different ways to average the model (1: model selection, 2: model selection with bootstrap OFV, 3: model average, 4: model average with bootstrap OFV)
 N_model=5 # number of models
 chaiSquare=0.05 # cutoff p-value of the likelihood ratio test
 checkIdentifiability=TRUE
 confidenceLevel=0.7 # predefined limit for the probability of achieving target endpoint
 modelsToInclude=c(TRUE, TRUE, TRUE, TRUE, TRUE)

## Read in the raw_result files

rawres_model0=read.csv("raw_results_estimation_model0.csv", header = TRUE, sep = ",")
 rawres_model1=read.csv("raw_results_estimation_model1.csv", header = TRUE, sep = ",")
 rawres_model2=read.csv("raw_results_estimation_model2.csv", header = TRUE, sep = ",")
 rawres_model3=read.csv("raw_results_estimation_model3.csv", header = TRUE, sep = ",")
 rawres_model4=read.csv("raw_results_estimation_model4.csv", header = TRUE, sep = ",")

 if (checkIdentifiability){
 # precond bootstrap raw_result files (optional used for the check for identifiability of the model parameters)
 precond_rawres_model0=read.csv("precond_raw_results_estimation_model0.csv", header = TRUE, sep = ",")
 precond_rawres_model1=read.csv("precond_raw_results_estimation_model1.csv", header = TRUE, sep = ",")
 precond_rawres_model2=read.csv("precond_raw_results_estimation_model2.csv", header = TRUE, sep = ",")
 precond_rawres_model3=read.csv("precond_raw_results_estimation_model3.csv", header = TRUE, sep = ",")
 precond_rawres_model4=read.csv("precond_raw_results_estimation_model4.csv", header = TRUE, sep = ",")

 precond_rawres_model0[1,1]=0
 precond_rawres_model1[1,1]=0
 precond_rawres_model2[1,1]=0
 precond_rawres_model3[1,1]=0
 precond_rawres_model4[1,1]=0
 }

## Define dose-endpoint relationship

Depending on the model and endpoint definition (e.g., if random effect variable is nonlinear with respect to the endpoint) we may need stochastical simulation/integration to compute endpoint as implemented in C++ version. The computaional speed of these functions will directly influence the overall computatioanl speed, so try to implement it as effiecient as possible if stochastical simulation is needed. For this example the end-point is the mean of the population and the random variable is linear with respect to the end point, we can simulate the endpoint just using fixed effect parameters.

dose_endpoint_sim_model0 <- function(dose_in){ #h_{0,j} in the manuscript PLACEBO MODEL
 endpoint_sim=0*dose_in
 return(endpoint_sim)
 }
 dose_endpoint_sim_model1 <- function(dose_in, slope ){ #h_{1,j} in the manuscript
 slope=abs(slope)
 endpoint_sim=slope*dose_in
 return(endpoint_sim)
 }
 dose_endpoint_sim_model2 <- function(dose_in, A ,M ){ #h_{2,j} in the manuscript
 A=abs(A)
 M=abs(M)
 endpoint_sim=M*log(1+A*dose_in)
 return(endpoint_sim)
 }
 dose_endpoint_sim_model3 <- function(dose_in, EMAX ,ED50 ){ #h_{3,j} in the manuscript
 EMAX=abs(EMAX)
 ED50=abs(ED50)
 endpoint_sim=EMAX*dose_in/(ED50+dose_in)
 return(endpoint_sim)
 }
 dose_endpoint_sim_model4 <- function(dose_in, EMAX ,ED50, N ){ #h_{4,j} in the manuscript
 EMAX=abs(EMAX)
 ED50=abs(ED50)
 N=abs(N)
 endpoint_sim=EMAX*dose_in^(N)/(ED50^(N)+dose_in^(N))
 return(endpoint_sim)
 }

## For each model, simulate the end point for each dose

The computational cost can be saved by only simulating the endpoint for the models that were selected

N_bootstrap=nrow(rawres_model0)
 N_dose=length(dose)
 endpoint_sim_model0=matrix(rep(NaN, N_bootstrap*N_dose),nrow=N_bootstrap,ncol=N_dose)
 endpoint_sim_model1=matrix(rep(NaN, N_bootstrap*N_dose),nrow=N_bootstrap,ncol=N_dose)
 endpoint_sim_model2=matrix(rep(NaN, N_bootstrap*N_dose),nrow=N_bootstrap,ncol=N_dose)
 endpoint_sim_model3=matrix(rep(NaN, N_bootstrap*N_dose),nrow=N_bootstrap,ncol=N_dose)
 endpoint_sim_model4=matrix(rep(NaN, N_bootstrap*N_dose),nrow=N_bootstrap,ncol=N_dose)

 for(j in 1:N_bootstrap){
 endpoint_sim_model0[j,]=dose_endpoint_sim_model0(dose)
 endpoint_sim_model1[j,]=dose_endpoint_sim_model1(dose, rawres_model1[j,"p_1"])
 endpoint_sim_model2[j,]=dose_endpoint_sim_model2(dose, rawres_model2[j,"p_1"], rawres_model2[j,"p_2"])
 endpoint_sim_model3[j,]=dose_endpoint_sim_model3(dose, rawres_model3[j,"EMAX"], rawres_model3[j,"ED50"])
 endpoint_sim_model4[j,]=dose_endpoint_sim_model4(dose, rawres_model4[j,"EMAX"], rawres_model4[j,"ED50"], rawres_model4[j,"gamma"])

 }

## Construct Probability of Success v.s. Dose relationship

Input endpoint simulation results for each model for each dose, [number of model] (N_bootstrap x N_dose) matricies Output Probability of success for each dose, N_dose vector

### Set the number of model parameters

If we decide not to use the model, we can set modelsToInclude[j] to be FALSE so that number of parameters will be set to be Infinity leading to zero weight using AIC.

if (modelsToInclude[1]){
 numPara_model0=0
 }else{
 numPara_model0=Inf
 }

 if(modelsToInclude[2]){
 numPara_model1=1
 }else{
 numPara_model1=Inf
 }

 if(modelsToInclude[3]){
 numPara_model2=2
 }else{
 numPara_model2=Inf
 }

 if(modelsToInclude[4]){
 numPara_model3=2
 }else{
 numPara_model3=Inf
 }

 if(modelsToInclude[5]){
 numPara_model4=3
 }else{
 numPara_model4=Inf
 }

### Calculate the weight using AIC, Likelihood Ratio test and Identifiability test.

Note weight will be in log scale for computational stability. We set weight to be zero (-inf in log scale) when the model fails LRT. We set weight to be zero (-inf in log scale) when the model fails identifiability test.

choice=rep(0,N_bootstrap)

 weights=matrix(rep(-Inf, N_bootstrap*N_model),nrow=N_bootstrap,ncol=N_model)
 weights_chai=matrix(rep(-Inf, N_bootstrap*N_model),nrow=N_bootstrap,ncol=N_model)

 identifyabilityCheckFlag=matrix(rep(0, N_bootstrap*N_model),nrow=N_bootstrap,ncol=N_model)

 # Weight using AIC
 for(j in 1:N_bootstrap){
 # w_j in manuscript

 ## AIC
 weights[j,]=c((rawres_model0[j,"ofv"]+2*numPara_model0)/(-2), (rawres_model1[j,"ofv"]+2*numPara_model1)/(-2), (rawres_model2[j,"ofv"]+2*numPara_model2)/(-2), (rawres_model3[j,"ofv"]+2*numPara_model3)/(-2), (rawres_model4[j,"ofv"]+2*numPara_model4)/(-2))


 if (chaiSquare>0){
 if(chaiSquare==0.05){
 weights_chai[j,]=c((rawres_model0[j,"ofv"]+2*numPara_model0)/(-2), (rawres_model1[j,"ofv"]+2*(numPara_model1-1)+3.84)/(-2), (rawres_model2[j,"ofv"]+2*(numPara_model2-2)+5.99)/(-2), (rawres_model3[j,"ofv"]+2*(numPara_model3-2)+5.99)/(-2), (rawres_model4[j,"ofv"]+2*(numPara_model4-3)+7.82)/(-2))
 for (k in 2:N_model){
 if (is.nan(weights_chai[j,k])|is.na(weights_chai[j,k])|is.na(weights_chai[j,1])|is.nan(weights_chai[j,1])){
 }else{
 if(weights_chai[j,k]<weights_chai[j,1]){
 weights[j,k]=-Inf;
 }
 }
 }
 }else if(chaiSquare==0.025){
 weights_chai[j,]=c((rawres_model0[j,"ofv"]+2*numPara_model0)/(-2), (rawres_model1[j,"ofv"]+2*(numPara_model1-1)+5.024)/(-2), (rawres_model2[j,"ofv"]+2*(numPara_model2-2)+7.378)/(-2), (rawres_model3[j,"ofv"]+2*(numPara_model3-2)+7.378)/(-2), (rawres_model4[j,"ofv"]+2*(numPara_model4-3)+9.348)/(-2))
 for (k in 2:N_model){
 if (is.nan(weights_chai[j,k])|is.na(weights_chai[j,k])|is.na(weights_chai[j,1])|is.nan(weights_chai[j,1])){
 }else{
 if(weights_chai[j,k]<weights_chai[j,1]){
 weights[j,k]=-Inf;
 }
 }
 }
 }else{
 stop('the chai square distribution for the p-value specified by the user is not defined in the code')
 }

 }

 choice[j]=which.max(weights[j,])
 }


 #Check the identifiability using precond bootstrap
 if(checkIdentifiability){

 paraDiffTol=0.10

 for(j in 1:N_bootstrap){

 # model1
 tempDF=precond_rawres_model1[precond_rawres_model1$model==(j-1),]

 if (nrow(tempDF)==1){
 # model0 (no need to check as this is a placebo model, i.e., no drug effect related parameter)

 if(!is.nan(abs(rawres_model1[j,"ofv"]-tempDF[1,"ofv"]))&!is.na(abs(rawres_model1[j,"ofv"]-tempDF[1,"ofv"]))){
 if(abs(rawres_model1[j,"ofv"]-tempDF[1,"ofv"])<0.1){ # give 0.1 ofv different tolerence as the numerically computed result cannot be exactly the same
 if((abs(rawres_model1[j,"p_1"])-abs(tempDF[1,"p_1"]))/(abs(rawres_model1[j,"p_1"])+abs(tempDF[1,"p_1"]))>paraDiffTol){
 weights[j,2]=-Inf #reject the model as SLOPE is not estimable
 }
 }else{
 identifyabilityCheckFlag[j,2]=2
 }
 }else{
 identifyabilityCheckFlag[j,2]=1
 }
 }else{
 identifyabilityCheckFlag[j,2]=3
 }


 # model2
 tempDF=precond_rawres_model2[precond_rawres_model2$model==(j-1),]

 if (nrow(tempDF)==1){
 if(!is.nan(abs(rawres_model2[j,"ofv"]-tempDF[1,"ofv"]))&!is.na(abs(rawres_model2[j,"ofv"]-tempDF[1,"ofv"]))){

 if(abs(rawres_model2[j,"ofv"]-tempDF[1,"ofv"])<0.1){ # give 0.1 ofv different tolerence as the numerically computed result cannot be exactly the same
 if((abs(rawres_model2[j,"p_1"])-abs(tempDF[1,"p_1"]))/(abs(rawres_model2[j,"p_1"])+abs(tempDF[1,"p_1"]))>paraDiffTol){
 weights[j,3]=-Inf #reject the model as A is not estimable
 }
 if((abs(rawres_model2[j,"p_2"])-abs(tempDF[1,"p_2"]))/(abs(rawres_model2[j,"p_2"])+abs(tempDF[1,"p_2"]))>paraDiffTol){
 weights[j,3]=-Inf #reject the model as M is not estimable
 }
 }else{
 identifyabilityCheckFlag[j,3]=2
 }
 }else{
 identifyabilityCheckFlag[j,3]=1
 }
 }else{
 identifyabilityCheckFlag[j,3]=3
 }

 # model3
 tempDF=precond_rawres_model3[precond_rawres_model3$model==(j-1),]

 if (nrow(tempDF)==1){
 if(!is.nan(abs(rawres_model3[j,"ofv"]-tempDF[1,"ofv"]))&!is.na(abs(rawres_model3[j,"ofv"]-tempDF[1,"ofv"]))){

 if(abs(rawres_model3[j,"ofv"]-tempDF[1,"ofv"])<0.1){ # give 0.1 ofv different tolerence as the numerically computed result cannot be exactly the same
 if((abs(rawres_model3[j,"EMAX"])-abs(tempDF[1,"EMAX"]))/(abs(rawres_model3[j,"EMAX"])+abs(tempDF[1,"EMAX"]))>paraDiffTol){
 weights[j,4]=-Inf #reject the model as EMAX is not estimable
 }
 if((abs(rawres_model3[j,"ED50"])-abs(tempDF[1,"ED50"]))/(abs(rawres_model3[j,"ED50"])+abs(tempDF[1,"ED50"]))>paraDiffTol){
 weights[j,4]=-Inf #reject the model as ED50 is not estimable
 }
 }else{
 identifyabilityCheckFlag[j,4]=2
 }
 }else{
 identifyabilityCheckFlag[j,4]=1
 }
 }else{
 identifyabilityCheckFlag[j,4]=3
 }

 # model4
 tempDF=precond_rawres_model4[precond_rawres_model4$model==(j-1),]

 if (nrow(tempDF)==1){
 if(!is.nan(abs(rawres_model4[j,"ofv"]-tempDF[1,"ofv"]))&!is.na(abs(rawres_model4[j,"ofv"]-tempDF[1,"ofv"]))){

 if(abs(rawres_model4[j,"ofv"]-tempDF[1,"ofv"])<0.1){ # give 0.1 ofv different tolerence as the numerically computed result cannot be exactly the same
 if((abs(rawres_model4[j,"EMAX"])-abs(tempDF[1,"EMAX"]))/(abs(rawres_model4[j,"EMAX"])+abs(tempDF[1,"EMAX"]))>paraDiffTol){

 weights[j,5]=-Inf #reject the model as EMAX is not estimable
 }
 if((abs(rawres_model4[j,"ED50"])-abs(tempDF[1,"ED50"]))/(abs(rawres_model4[j,"ED50"])+abs(tempDF[1,"ED50"]))>paraDiffTol){
 weights[j,5]=-Inf #reject the model as ED50 is not estimable
 }
 if((abs(rawres_model4[j,"gamma"])-abs(tempDF[1,"gamma"]))/(abs(rawres_model4[j,"gamma"])+abs(tempDF[1,"gamma"]))>paraDiffTol){
 weights[j,5]=-Inf #reject the model as N is not estimable
 }
 }else{
 identifyabilityCheckFlag[j,5]=2
 }
 }else{
 identifyabilityCheckFlag[j,5]=1
 }
 }else{
 identifyabilityCheckFlag[j,5]=3
 }
 choice[j]=which.max(weights[j,])
 }
 }

 for(j in 1:N_bootstrap){
 cumWeight=1
 for(i in 2:N_model){
 if ((!is.nan(weights[j,i]))&&(!is.na(weights[j,i]))){
 cumWeight=cumWeight+exp(weights[j,i]-weights[j,1]);
 }
 }
 tempBase=weights[j,1];
 for(i in 1:N_model){
 if ((!is.nan(weights[j,i]))&&(!is.na(weights[j,i]))){
 weights[j,i]=exp(weights[j,i]-tempBase)/cumWeight
 }else{
 weights[j,i]=0
 }
 }
 }

### Calculate the probability of achieving target end point.

averaged_endpoint_sim=matrix(N_bootstrap*length(dose),nrow=N_bootstrap,ncol=length(dose))
 chosenIndex=rep(NaN,N_bootstrap)
 PoS=rep(0,N_dose)
 bootstrapSampleOK=rep(0,N_bootstrap)

 for(i in 1:N_bootstrap){
 if (!is.na(sum(weights[i,]))&&!is.nan(sum(weights[i,]))){
 if (round(sum(weights[i,])*1000)==1000){
 bootstrapSampleOK[i]=1
 }
 }
 }

 if (method==1){ # model selection
 chosenIndex=rep(which.max(weights[1,]),N_bootstrap)

 for(i in 1:N_bootstrap){
 if (bootstrapSampleOK[i]){
 if (chosenIndex[i]==1){
 averaged_endpoint_sim[i,]=endpoint_sim_model0[i,]
 }else if (chosenIndex[i]==2){
 averaged_endpoint_sim[i,]=endpoint_sim_model1[i,]
 }else if (chosenIndex[i]==3){
 averaged_endpoint_sim[i,]=endpoint_sim_model2[i,]
 }else if (chosenIndex[i]==4){
 averaged_endpoint_sim[i,]=endpoint_sim_model3[i,]
 }else if (chosenIndex[i]==5){
 averaged_endpoint_sim[i,]=endpoint_sim_model4[i,]
 }else if (chosenIndex[i]==6){
 averaged_endpoint_sim[i,]=endpoint_sim_model5[i,]
 }
 }
 }

 for(l in 1:N_dose){
 for(i in 1:N_bootstrap){
 if (bootstrapSampleOK[i]){
 if (!is.na(averaged_endpoint_sim[i,l])&&!is.nan(averaged_endpoint_sim[i,l])){
 if(averaged_endpoint_sim[i,l]>TV){
 PoS[l]=PoS[l]+1
 }
 }
 }
 }
 PoS[l]=PoS[l]/sum(bootstrapSampleOK)
 }

 }else if(method==2){ # model selection with bootstrap OFV

 for(i in 1:N_bootstrap){
 if (bootstrapSampleOK[i]){
 chosenIndex[i]=which.max(weights[i,])

 if (chosenIndex[i]==1){
 averaged_endpoint_sim[i,]=endpoint_sim_model0[i,]
 }else if (chosenIndex[i]==2){
 averaged_endpoint_sim[i,]=endpoint_sim_model1[i,]
 }else if (chosenIndex[i]==3){
 averaged_endpoint_sim[i,]=endpoint_sim_model2[i,]
 }else if (chosenIndex[i]==4){
 averaged_endpoint_sim[i,]=endpoint_sim_model3[i,]
 }else if (chosenIndex[i]==5){
 averaged_endpoint_sim[i,]=endpoint_sim_model4[i,]
 }else if (chosenIndex[i]==6){
 averaged_endpoint_sim[i,]=endpoint_sim_model5[i,]
 }
 }
 }
 for(l in 1:N_dose){
 for(i in 1:N_bootstrap){
 if (bootstrapSampleOK[i]){
 if(averaged_endpoint_sim[i,l]>TV){
 PoS[l]=PoS[l]+1
 }
 }
 }
 PoS[l]=PoS[l]/sum(bootstrapSampleOK)
 }
 }else if(method==3){ # model average
 for (l in 1:N_dose){
 for(i in 1:N_bootstrap){
 if (bootstrapSampleOK[i]){
 #model 0
 if (!is.na(endpoint_sim_model0[i,l])&&!is.nan(endpoint_sim_model0[i,l])){
 if(endpoint_sim_model0[i,l]>TV){
 PoS[l]=PoS[l]+weights[1,1]/sum(bootstrapSampleOK)
 }
 }
 #model 1
 if (!is.na(endpoint_sim_model1[i,l])&&!is.nan(endpoint_sim_model1[i,l])){
 if(endpoint_sim_model1[i,l]>TV){
 PoS[l]=PoS[l]+weights[1,2]/sum(bootstrapSampleOK)
 }
 }
 #model 2
 if (!is.na(endpoint_sim_model2[i,l])&&!is.nan(endpoint_sim_model2[i,l])){
 if(endpoint_sim_model2[i,l]>TV){
 PoS[l]=PoS[l]+weights[1,3]/sum(bootstrapSampleOK)
 }
 }
 #model 3
 if (!is.na(endpoint_sim_model3[i,l])&&!is.nan(endpoint_sim_model3[i,l])){
 if(endpoint_sim_model3[i,l]>TV){
 PoS[l]=PoS[l]+weights[1,4]/sum(bootstrapSampleOK)
 }
 }
 #model 4
 if (!is.na(endpoint_sim_model4[i,l])&&!is.nan(endpoint_sim_model4[i,l])){
 if(endpoint_sim_model4[i,l]>TV){
 PoS[l]=PoS[l]+weights[1,5]/sum(bootstrapSampleOK)
 }
 }
 }
 }
 }
 }else if(method==4){ # model average with bootstrap OFV
 for (l in 1:N_dose){
 for(i in 1:N_bootstrap){
 if (bootstrapSampleOK[i]){
 #model 0
 if (!is.na(endpoint_sim_model0[i,l])&&!is.nan(endpoint_sim_model0[i,l])){
 if(endpoint_sim_model0[i,l]>TV){
 PoS[l]=PoS[l]+weights[i,1]/sum(bootstrapSampleOK)
 }
 }
 #model 1
 if (!is.na(endpoint_sim_model1[i,l])&&!is.nan(endpoint_sim_model1[i,l])){
 if(endpoint_sim_model1[i,l]>TV){
 PoS[l]=PoS[l]+weights[i,2]/sum(bootstrapSampleOK)
 }
 }
 #model 2
 if (!is.na(endpoint_sim_model2[i,l])&&!is.nan(endpoint_sim_model2[i,l])){
 if(endpoint_sim_model2[i,l]>TV){
 PoS[l]=PoS[l]+weights[i,3]/sum(bootstrapSampleOK)
 }
 }
 #model 3
 if (!is.na(endpoint_sim_model3[i,l])&&!is.nan(endpoint_sim_model3[i,l])){
 if(endpoint_sim_model3[i,l]>TV){
 PoS[l]=PoS[l]+weights[i,4]/sum(bootstrapSampleOK)
 }
 }
 #model 4
 if (!is.na(endpoint_sim_model4[i,l])&&!is.nan(endpoint_sim_model4[i,l])){
 if(endpoint_sim_model4[i,l]>TV){
 PoS[l]=PoS[l]+weights[i,5]/sum(bootstrapSampleOK)
 }
 }
 }
 }
 }
 }

## Dose selection

Input probability of success: N_dose vector Output Selected dosage: a scalar value

PoMED=rep(NaN, N_dose)
 for(l in 1:N_dose-1){
 PoMED[l]=PoS[l+1]-PoS[l]
 }

 PoMED[N_dose]=1-PoS[N_dose]
 SelectedDose=NaN
 prob=0
 for(l in N_dose:1){
 if(PoS[l]>confidenceLevel){
 SelectedDose=dose[l]
 prob=PoS[l]
 }
 }

 meanWeights=rep(NaN, ncol(weights))
 for (l in 1: ncol(weights)){
 meanWeights[l]=mean(weights[,l])
 }

 outputList=list(SelectedDose=SelectedDose,probabilityMED=prob, PoMED=PoMED, meanWeights=meanWeights, endpoint_sim_model0=endpoint_sim_model0,endpoint_sim_model1=endpoint_sim_model1,endpoint_sim_model2=endpoint_sim_model2,endpoint_sim_model3=endpoint_sim_model3,endpoint_sim_model4=endpoint_sim_model4, PoS=PoS, averaged_endpoint_sim=averaged_endpoint_sim, weights=weights)
 print(paste("The minimum dose estimated to achieve target endpoint with more than " ,confidenceLevel*100, "% of probability:",outputList$SelectedDose,"mg"))

## [1] "The minimum dose estimated to achieve target endpoint with more than 70 % of probability: 400 mg"

## Probability of achieving endpoint v.s. dose plot

library(ggplot2)

 # Dose-Probability of achieving endpoint plot
 PoSPlot=data.frame(Dose=dose,Probability_of_achieving_endpoint=outputList$PoS)
 ggplot(PoSPlot, aes(x=Dose, y=Probability_of_achieving_endpoint*100))+
 geom_vline(xintercept = 10,color='white',size=1)+
 geom_vline(xintercept = 40,color='white',size=1)+
 geom_vline(xintercept = 100,color='white',size=1)+
 geom_vline(xintercept = 400,color='white',size=1)+
 geom_point()+geom_line()+
 geom_hline(yintercept = confidenceLevel*100,color=rgb(153/255,0,0),size=1)+
 scale_x_continuous(
 breaks = c(0, 10, 40, 100, 400),
 label = c("","10mg", "40mg", "100mg","400mg"),
 minor_breaks=NULL
 )+
 scale_y_continuous(
 breaks = c(0, 25, 50, 70, 75, 100),
 minor_breaks=NULL
 )+ylab("Probability of achieving target endpoint (%)")


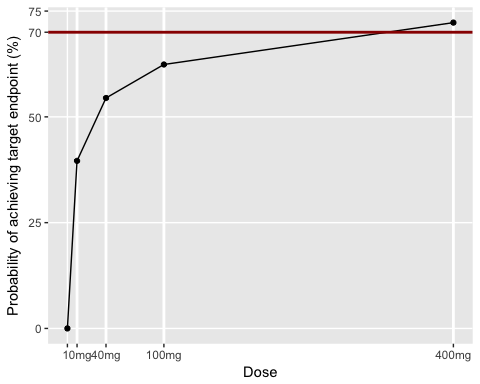


## Simulated endpoint histogram for Method 2 (Bootstrap model selection)

histoPlot_df=data.frame(averagedEndpointSim=outputList$averaged_endpoint_sim[,2], Dose="10 mg")
 histoPlot_df=rbind(histoPlot_df, data.frame(averagedEndpointSim=outputList$averaged_endpoint_sim[,3], Dose="40 mg"))
 histoPlot_df=rbind(histoPlot_df, data.frame(averagedEndpointSim=outputList$averaged_endpoint_sim[,4], Dose="100 mg"))
 histoPlot_df=rbind(histoPlot_df, data.frame(averagedEndpointSim=outputList$averaged_endpoint_sim[,5], Dose="400 mg"))

 ggplot(histoPlot_df, aes(averagedEndpointSim))+geom_histogram(binwidth=0.025)+ylim(c(0,50))+xlim(c(-0.05, 0.35))+ geom_vline(xintercept = 0.1)+facet_grid(.~Dose)+
 xlab("Simulated Endpoint")+ylab("Count")

## Warning: Removed 8 rows containing missing values (geom_bar).


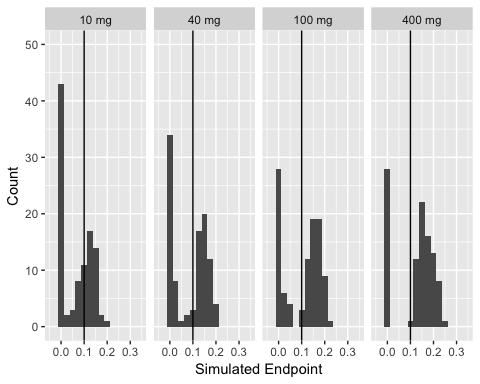

Supplement: Supplementary file 11 — Supplementary material 11 (DOCX 62 kb) [file 10928_2017_9550_MOESM11_ESM.docx]
